# A group-based behavioural intervention for weight management (PROGROUP) versus usual care in adults with severe obesity: a feasibility randomised controlled trial protocol

**DOI:** 10.1186/s40814-022-01167-0

**Published:** 2022-09-10

**Authors:** Dawn Swancutt, Mark Tarrant, Wendy Ingram, Sarah Baldrey, Lorna Burns, Richard Byng, Raff Calitri, Siobhan Creanor, Sarah Dean, Lucy Evans, Laura Gill, Elizabeth Goodwin, Lily Hawkins, Chris Hayward, Sarah Hind, Laura Hollands, Joanne Hosking, Jenny Lloyd, Shokraneh Moghadam, Helen Neilens, Mary O’Kane, Steve Perry, Rod Sheaff, Anne Spencer, Adrian Taylor, Thomas Ward, Ross Watkins, John Wilding, Jonathan Pinkney

**Affiliations:** 1 University of Plymouth, Faculty of Health, University of Plymouth, Plymouth Science Park, Plymouth, PL6 8BX UK; 2 University of Exeter, College of Medicine and Health, St Luke’s Campus, Heavitree Road, Exeter, EX1 2LU UK; 3 Livewell South West, Cumberland Centre, Damerel Close, Devonport, Plymouth, PL1 4JZ UK; 4 University Hospitals Plymouth NHS Trust, Bircham Park Offices, 1 Roscoff Rise, Derriford, Plymouth, PL6 5FP UK; 5 Leeds Teaching Hospital NHS Trust, St James’s University Hospital, Beckett Street, Leeds, LS9 7TF UK; 6 Independent Consultant, Plymouth, UK; 7 University of Liverpool, Department of Cardiovascular and Metabolic Medicine, Clinical Sciences Centre, Aintree University Hospital, Longmoor Lane, Liverpool, L9 7AL UK

**Keywords:** Severe obesity, Weight management, Group-based, Feasibility, Behavioural intervention

## Abstract

**Background:**

Approximately 15 million people in the UK live with obesity, around 5 million of whom have severe obesity (body mass index (BMI) ≥35kg/m^2^). Having severe obesity markedly compromises health, well-being and quality of life, and substantially reduces life expectancy. These adverse outcomes are prevented or ameliorated by weight loss, for which sustained behavioural change is the cornerstone of treatment. Although NHS specialist ‘Tier 3’ Weight Management Services (T3WMS) support people with severe obesity, using individual and group-based treatment, the current evidence on optimal intervention design and outcomes is limited. Due to heterogeneity of severe obesity, there is a need to tailor treatment to address individual needs. Despite this heterogeneity, there are good reasons to suspect that a structured group-based behavioural intervention may be more effective and cost-effective for the treatment of severe obesity compared to usual care. The aims of this study are to test the feasibility of establishing and delivering a multi-centre randomised controlled clinical trial to compare a group-based behavioural intervention versus usual care in people with severe obesity.

**Methods:**

This feasibility randomised controlled study is a partially clustered multi-centre trial of PROGROUP (a novel group-based behavioural intervention) versus usual care. Adults ≥18 years of age who have been newly referred to and accepted by NHS T3WMS will be eligible if they have a BMI ≥40, or ≥35 kg/m^2^ with comorbidity, are suitable for group-based care and are willing to be randomised. Exclusion criteria are participation in another weight management study, planned bariatric surgery during the trial, and unwillingness or inability to attend group sessions. Outcome assessors will be blinded to treatment allocation and success of blinding will be evaluated. Clinical measures will be collected at baseline, 6 and 12 months post-randomisation. Secondary outcome measures will be self-reported and collected remotely. Process and economic evaluations will be conducted.

**Discussion:**

This randomised feasibility study has been designed to test all the required research procedures and additionally explore three key issues; the feasibility of implementing a complex trial at participating NHS T3WMS, training the multidisciplinary healthcare teams in a standard intervention, and the acceptability of a group intervention for these particularly complex patients.

**Trial registration:**

ISRCTN number 22088800.

**Supplementary Information:**

The online version contains supplementary material available at 10.1186/s40814-022-01167-0.

## Background

### The challenge of treating severe obesity

Approximately 15 million people in the UK are living with obesity, of whom at least 5 million have severe obesity (body mass index (BMI) ≥35kg/m^2^) [1]. At higher levels of BMI (e.g. 40–45kg/m^2^), which are commonly seen in NHS specialist weight management services, an average loss of life expectancy is 8–10 years [2], meaning that approximately 1.5 million adults in the UK face early death attributable to this condition—and meanwhile, are living with substantially compromised psychosocial health, well-being and quality of life [3, 4]. With recent estimates predicting a dramatic rise in obesity prevalence, the UK All Party Parliamentary Group describes obesity as ‘a problem the country cannot afford to defer to the next generation’ [5].

Currently, the annual cost of obesity to the UK national health service (NHS) is estimated to be between £2.47 [6] billion and £5.1 billion [7], and this is predicted to rise to £10 billion by 2050. The wider costs to society are even greater—estimated at £50 billion [8]. Societal impacts are felt through higher levels of worker absenteeism, reducing productivity [9], increased dependence on disability benefits, early retirement, increased levels of chronic disease [10] and mental health concerns [11] arising from obesity-related experiences such as weight stigma [12]. Although all sections of society are at risk of obesity, those of lower socioeconomic status, as well as ethnic minorities, experience a greater burden [7].

The World Obesity Federation highlighted the global impact of obesity and how most of its detrimental effects are prevented by weight loss [13]. However, severe obesity is a complex condition that is influenced by psychological, social and environmental factors, including childhood trauma, stigma and mental health problems, socioeconomic status, and availability (and marketing) of unhealthy foods [8, 14]. Apart from bariatric surgery (which relatively few people access), the treatment options for people with severe obesity (PWSO) are variable and of uncertain effectiveness [15]. Although the NHS specialist ‘Tier 3’ Weight Management Services (T3WMS) to support PWSO, the optimum design for treatments/interventions remains poorly defined, and there is little evidence on what outcomes treatment has [16, 17].

### Group-based behavioural interventions

The potential of group interventions to support the treatment of long-term conditions is already recognised [18, 19]. Group-based treatment may also allow service capacity to be scaled-up, when faced with large patient numbers. Key psychosocial resources linked to behaviour change, including social support and self-efficacy [20], can be triggered when group members form a sense of social connectedness, or *shared social identity*, with other group members [21, 22]. Being socially connected to others is associated with improvement in a variety of health outcomes, including a reduction in mortality rate [23]. Indeed, social connectedness appears to be a stronger predictor of a lower mortality rate than other well-known risk factors such as excessive alcohol consumption and smoking [24]. A recent systematic review and meta-analysis confirmed that interventions which specifically build shared social identity have a moderate-to-strong impact on a range of health outcomes, including psychosocial, cognitive, mental and physical health (standardised effect size of 0.66) [22]. Groups are increasingly used to deliver behaviour change interventions in healthcare, but there is a need for more UK-centred evaluation and clearer delineation of group delivery components [18, 19].

### Group-based behavioural interventions in the T3WMS setting

Effective behavioural intervention is a fundamental requirement for successful weight management. Our recent review of randomised controlled trials (RCTs) of group-based behavioural interventions reported superior weight loss at 6 months in group interventions versus controls (mean weight loss difference = −3.6 kg, 95% CI [-4.15, −2.84]), with effects persisting up to 24 months (mean weight loss difference = −2.56 kg, 95% CI [−3.79, −1.33]) [25]. The mean baseline BMI of the participants in the included RCTs was only 33.8kg/m^2^ (range 29.3–41.0), a lower weight category than that of severe obesity, and so generalisability to PWSO is unclear. The ‘Look AHEAD’ trial in the USA also included a group intervention for weight loss (mean baseline BMI = 36kg/m^2^) [26], but it is uncertain whether findings extrapolate to other populations, including the UK and PWSO.

Group interventions may enhance treatment for PWSO by contributing health benefits beyond the efficient delivery of programme content [18, 19, 22, 27, 28]. In our recent evaluation of a UK group-based T3WMS weight loss programme, patients reported how the shared social identity formed within the treatment setting was a key mechanism, structuring their engagement with and progression through the group programme [29]. The development of group processes, and shared social identity specifically, is not currently considered in the design of T3WMS group programmes and is consequently neglected in practice [19]. Without active facilitation, group processes may cause interpersonal conflicts, or encourage the formation of group norms and cognitions that undermine behaviour change techniques [30] that might otherwise be effective [20]. Our recent research provides an evidence-base for understanding how to assemble groups in clinical settings and capitalise on their therapeutic potential [25, 28, 29], but there is a need for UK-centred evaluation of group delivery in T3WMS [19, 31].

In summary, several studies suggest the potential effectiveness for group-based behavioural intervention in T3WMS. It may also be possible to scale-up capacity in routine T3WMS by using group-based interventions to offer treatment to many more people than can be achieved with traditional care models. However, PWSO are under-represented in published research, and past studies preceded new evidence on best practice for developing group-based interventions [28, 32]. Therefore, it remains uncertain whether the adoption of group-based intervention in T3WMS would enhance patient outcomes and cost-effectiveness. The PROGROUP programme aims to address these challenges, by establishing the evidence needed to show what effects the intervention has, and if warranted by these effects, for the successful implementation of a new group-based behavioural intervention (‘PROGROUP’) for PWSO in T3WMS. This protocol describes a multi-centre randomised controlled trial with a parallel process evaluation to assess the feasibility and patient acceptability, and an economic evaluation of resource use tools, to inform the design of a future definitive RCT.

### Study objectives

To inform the design and delivery of a definitive RCT to compare the effectiveness and cost-effectiveness of the PROGROUP intervention compared with usual care, this randomised feasibility trial has the following objectives:

#### Trial feasibility objectives

- Estimate rates of screening, enrolment, recruitment, randomisation and retention
- Ascertain adherence to the intervention and usual care
- Ascertain completeness of participant-reported and clinical data collection

#### Process evaluation objectives

- Ascertain the feasibility and acceptability of outcome measures, trial processes, intervention and training package
- Ascertain the fidelity to intervention form and function and training delivery
- Understand mechanisms of action of PROGROUP (including shared social identity and related group processes)
- Assess contamination potential and organisational and policy contexts

#### Economic evaluation objectives

- Cost the intervention
- Pilot data collection methods/instruments for resource use, health-related quality of life (QOL) and well-being
- Piloting the cost-effectiveness modelling framework that will be used in the main trial

### Outcome measures

Outcomes collected in this feasibility trial are listed below in Table 1 (further detail in Additional file 1). They may be revised for a future definitive trial, as informed by this feasibility study and input from the Patient Advisory Group. The feasibility study will determine the ability to collect the planned participant data items successfully.

**Table 1:**
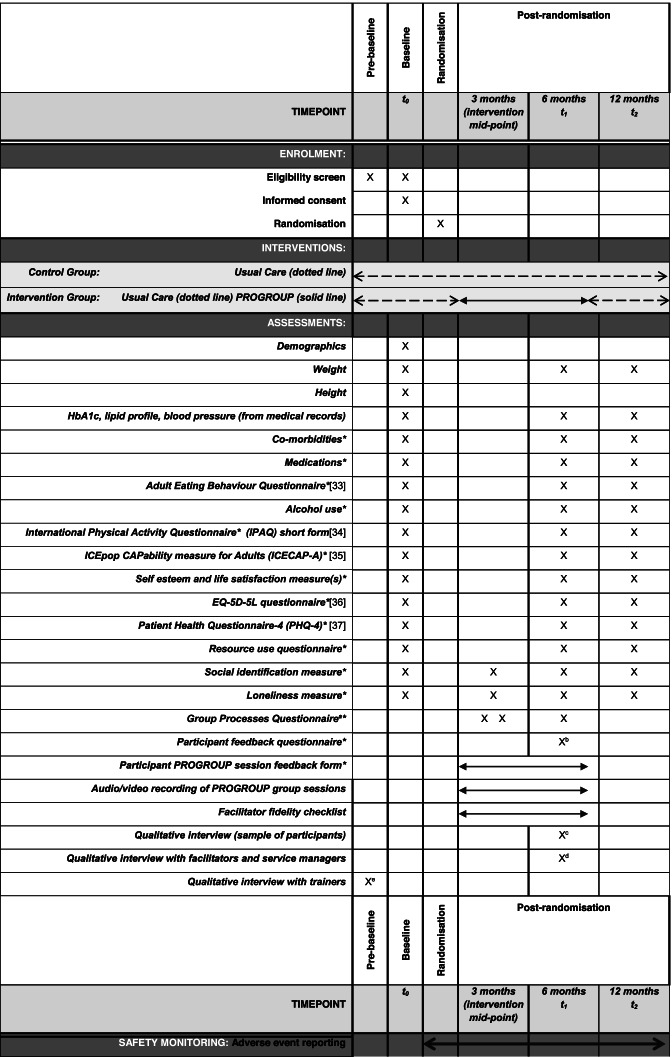
Study schedule

*Self-reported

^a^Following group sessions 2, 7 and 12

^b^Within a month of the 6-month time-point

^c^On completion of the intervention period

^d^Within a month after delivery of intervention

^e^Within a month after completion of training

Validated instruments referenced [33–37]

### Trial treatments

#### Usual care

Usual care will be an existing T3WMS programme consistent with the principles of NICE guideline CG189 [38] (sections 1.5 and 1.6, multidisciplinary behavioural change management delivered by appropriately trained professionals).

Group education activities that are sometimes used in usual care (e.g. introduction meetings, healthy eating education, activity sessions, bariatric education) will be permitted. However, deliberate, facilitated, group-based management of behaviour change, which is the defining feature of the PROGROUP intervention, is not present in the great majority of usual care interventions and is not permitted in this study.

It is expected that in this pragmatic feasibility trial there will be some variation in the structure and delivery of usual care across participating sites; usual care will be documented as part of the study. It is proposed that three sites will give a range of service contexts for evaluation.

#### Intervention ‘PROGROUP’

PROGROUP is a manualised intervention informed by the social identity approach to health [27], and social identity model of behaviour change specifically [28]. It is provided to participants in accordance with the manual by trained facilitators from a multi-disciplinary team (including nurses, dieticians, and physiotherapists) at each participating site.

In summary, the PROGROUP intervention consists of 15 contact sessions in total, over five months, as follows:

- (Weeks 1–2) Initial meeting with a facilitator (one-to-one).
- (Weeks 3–10) Eight consecutive, weekly group sessions.
- (Weeks 11–12) Interim one-to-one meeting to review of progress, including potential goal revision.
- (Weeks 14–20) Four consecutive, fortnightly group sessions: This part of the programme reflects a ‘behavioural maintenance’ phase.
- (Weeks 21–22) Final one-to-one session.

Each PROGROUP group consists of approximately 12 participants on average (expected range 8–15 participants). A group session takes approximately 2 h, including a scheduled break, and one-to-one sessions take approximately one hour each. The intervention is planned for face-to-face (in person) delivery but will transfer to remote (online) delivery if necessary, reflecting best practice in COVID response situations.

Referrals (e.g. to a psychologist) within the multi-disciplinary team can occur at any point within the programme based on participant need.

Whilst no ‘minimum dose’ for PROGROUP has been established, all participants (both trial arms) are asked to make every reasonable effort to adhere to their programme schedule. The importance of engagement with all trial activities is emphasised in the Participant Information Sheet.

## Methods/design

This is a multi-centre, partially clustered, feasibility randomised controlled trial of PROGROUP (group-based intervention) versus usual care (control). Details of which are reported according to the SPIRIT checklist, Additional file 2.

### Site eligibility criteria

**Fig. 1:**
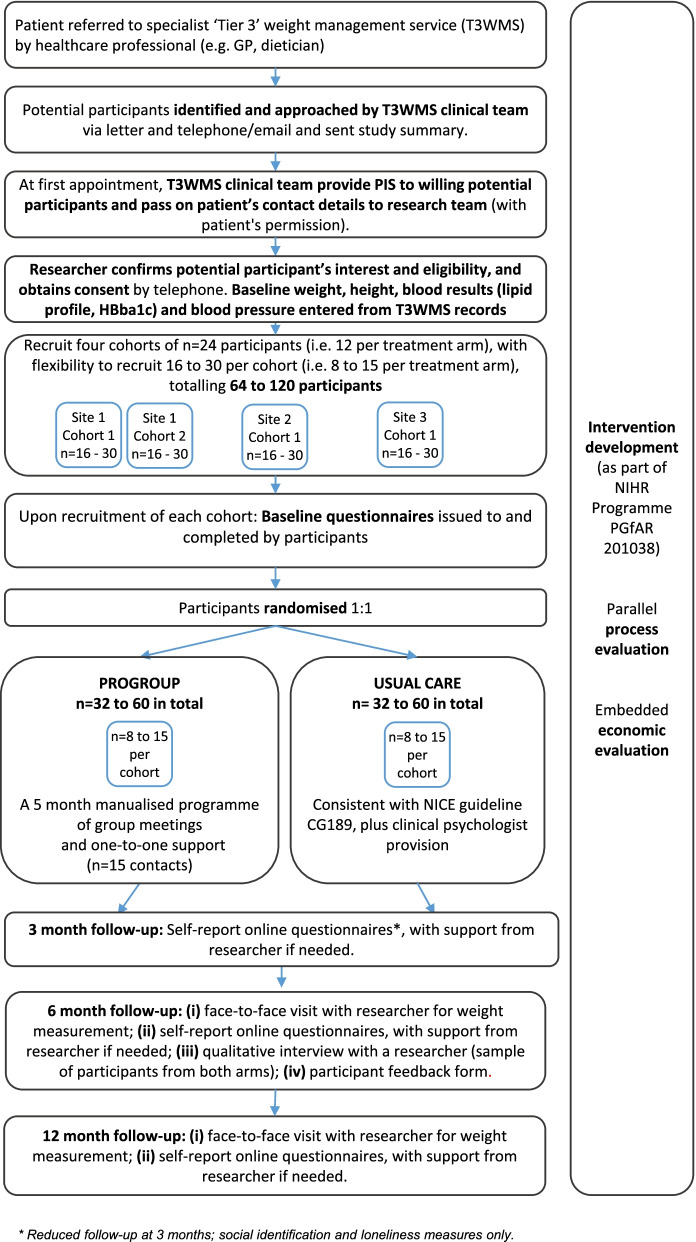
Study flow chart

The study aims to include three sites; running one group at each site, with one site running a second group (Fig. 1). Sites must meet the usual care criteria above, have a sufficient referral rate (or waiting list size) to be able to recruit patients within the study period, and have a multidisciplinary T3WMS team with the capacity to run the PROGROUP sessions.

### Participant eligibility criteria

#### Inclusion criteria

Patients must satisfy all of the following criteria to be enrolled in the study:

- Body mass index ≥40 or ≥35 kg/m^2^ with comorbidity
- Aged ≥18 years
- Willing to be randomised to either PROGROUP or usual care
- Registered with the participating T3WMS and considered suitable for group-based care.

#### Exclusion criteria

Patients who meet any of the following criteria will be excluded from participation:

- Currently engaged in any other weight management trial
- Have already had or are scheduled to undergo bariatric surgery during the course of the trial
- Unwilling or unable to attend group sessions
- Intending to relocate outside the geographical region during the trial period
- Participants who have significant difficulties such that they are unable to sufficiently understand/access the trial documentation or engage in group sessions.

Whist online data capture is the preferred means of collecting self-report outcome measures, alternative methods (postal, telephone) will be made available as needed. As such, lack of access to online services is not grounds for exclusion.

### Recruitment and consent

Site Principal Investigators will be responsible for promoting the study locally. Recruitment at each site will be closely monitored by the Trial Management Group (TMG). The stages of the recruitment process are summarised in Fig. 1: Study flow chart.

### Participant identification and initial approach

In all cases, identification of and approach to potential participants, including the provision of an initial study summary sheet, and the Patient Information Sheet (PIS), will be undertaken by the local site clinical and research staff.

Patients’ weight and height, lipid profile, systolic blood pressure and glycaemia status (measured as Hba1c) will be measured and recorded at the initial visit as part of the usual local T3WMS and used for informing eligibility assessment. If face-to-face appointments at the T3WMS are not in use, during COVID recovery, the initial visit may be conducted remotely (online or telephone). Alternative arrangements to measure weight and height will be offered in this scenario, to derive BMI for assessment against the inclusion criteria for BMI. Such alternatives include T3WMS staff obtaining the most recent weight and height measurement from primary care providers; researchers conducting a home visit and measuring weight and height using calibrated scales; accepting a patient-reported weight and height. Operational details for these alternative methods will be described in a study-specific work instruction. For the purposes of the study, this constitutes the ‘provisional’ baseline weight, height and BMI, used to determine eligibility.

Patients will give written permission for their contact details to be passed to the University consent team.

### Eligibility confirmation and consent

All patients will be given at least 24 h to consider the PIS before being contacted by members of the PROGROUP research team, to allow the opportunity to ask further questions about the study. The University researchers will conduct eligibility confirmation and the informed consent process remotely by telephone and send a copy of the consent form by e-mail (by post if preferred) to each participant. All members of the research team University research team have been trained in the relevant principles of Good Clinical Practice and the requirements of the trial protocol.

### Collection of clinical baseline measures

The researchers will update the study database to confirm that the patient is eligible and has provided informed consent. T3WMS staff will be notified at this point and asked to provide baseline clinical measures of height, weight, blood lipid profile (total cholesterol, HDL cholesterol, triglycerides), systolic blood pressure, and glycaemia measurement (HbA1c).

Should a routine face-to-face initial service appointment not be possible, clinical measures for all trial participants (PROGROUP and usual care groups) will be made at the same time, and before any treatment starts. For the purposes of the study, this measurement set constitutes the ‘measured’ baseline weight, height and BMI. Should a participant be found at this point to have a BMI that falls outside of the inclusion criteria for the study, the participant shall be withdrawn from the study.

### Recruiting complete cohorts of participants

The group-based nature of the PROGROUP intervention (i.e. partial clustering) necessitates the confirmed recruitment of a sufficient number of participants within a recruiting site prior to randomisation. As such, each site will aim to recruit a pool (‘cohort’) of ~24 participants, with flexibility to recruit 16–30, to be randomised at a single time-point.

In order to minimise the variable delay between the participants’ completion of self-reported baseline assessments and the point of randomisation, non-clinical, baseline assessments will be issued to participants for self-completion after a cohort is recruited.

Recruitment will continue as described until a cohort of participants is declared complete. The decision to declare a cohort complete will be made by the Co-Chief Investigators. Declaration of a completely recruited cohort will trigger the issue of baseline self-report assessments to participants.

It is envisaged that the time from eligibility assessment and consent to point of randomisation will be less than 4 weeks. This time interval will be monitored closely, including the factors that influence it, through central data monitoring and contact with participants. In the event that the interval exceeds 4 weeks, baseline weight will be rechecked prior to proceeding with randomisation.

### Recording screening and recruitment information

Accurate records of the recruitment process, required to meet the feasibility objectives of the study, will be maintained and reported as follows:

### Site records

Numbers of patients registered at T3WMS and the number provided with Study Summary Leaflets.

Number and characteristics of patients: provided with Participant Information Sheets; approached by research team; deemed ineligible (with reasons where available); declining to give consent (with reasons where available).

### Randomisation

After all baseline data collection is complete, participants will be randomly allocated to either the intervention (PROGROUP) or the control group (usual care), using a web-based block simultaneous randomisation system provided by the PenCTU.

Confirmation that randomisation has been performed will be communicated in a blinded fashion to local site staff and the research programme manager.

Further automatically generated emails will be sent to the site Principal Investigator and facilitators at the relevant site, disclosing the treatment group to which the participant has been allocated.

### Blinding

Participants are non-blinded, as it is not possible to conceal the treatment allocation from them. The outcome assessors (i.e. research team members conducting follow-up) will be blinded to treatment allocation. The success of outcome assessor-blinding will be evaluated by asking assessors to record the treatment group to which they think a participant has been allocated in the case report form, together with any instances of inadvertent unblinding (e.g. as a result of the participant disclosing their allocated treatment).

The initial data export provided to the trial statisticians will not disclose the treatment allocations, so that the analyses of the participant-reported outcomes, as well as the recruitment and retention rates, are blinded. In the event that the Programme Steering Committee (PSC) requests unblinded or disaggregated data during the trial, in order to fulfil its data monitoring duties, members of the PenCTU not involved in the conduct of the trial will assist with preparation of the data and transmission to PSG members, to maintain blinding of the trial statisticians and researchers involved in supporting patients to complete outcome measures.

### Process evaluation

In line with the MRC guidance on complex interventions [39], this study will include a realist mixed methods process evaluation that focuses on intervention mechanisms and implementation contexts. This will allow for the exploration of the acceptability and feasibility of implementing a national trial by assessing uptake (recruitment) and retention, participant engagement, fidelity of delivery (to form and function) and experiences of participants (patients, facilitators, managers). Any contamination effects, such as PROGROUP materials being used for control arm participants, and unintended consequences across trial arms, as well as any organisational contexts influencing delivery at each site, will be identified and documented. Qualitative data from interviews will be analysed using inductive thematic analysis for each interview group (participants, facilitators, trainers and service managers) using the Framework approach [40] to organise and code the data. A context-mechanism-outcome framework [41], will be supplemented with any other themes emerging directly from the data during coding. The generated themes will be used to update the logic model of the intervention. Any unintended consequences of the intervention and/or trial processes will be captured and synthesised and fed into revising trial processes, programme and training manuals. Any audio/video data relating to fidelity of delivery, participant engagement and contamination effects will be summarised into the framework to complement the qualitative data in the Framework Analysis. Quantitative data from questionnaires and checklists will be used to carry out a normative evaluation comparing the intervention that was delivered with what the PROGROUP model stipulates as well as to compare PROGROUP with usual care in terms of the trial feasibility and process evaluation objectives. Descriptive statistics will be used to summarise these data. Any free text data will be content-analysed, and the findings summarised into the framework to complement the qualitative data in the Framework Analysis.

### Economic evaluation

The economic evaluation will compare the economic costs and outcomes associated with the PROGROUP intervention compared to usual care in the feasibility trial, taking an NHS and Personal Social Services perspective, consistent with the methods recommended by the National Institute of Health and Care Excellence [42]. A further analysis will incorporate a societal perspective and the wider impacts on patients.

#### Intervention costings

The resource used for training and delivering the PROGROUP intervention (preparation, delivery, travel time) will be collected from the trainers and service managers, and nationally applicable unit costs will be applied. The feasibility study includes a cost analysis in order to pilot data collection and analytical procedures ahead of the main study. The results from this trial-based analysis will be presented in a disaggregated form, by the main cost drivers. Uncertainty in the intervention costs will be explored using sensitivity analysis.

#### Resource use and economic outcome measures

Data collection methods for primary, secondary and social care resource use, as well as personally incurred expenses (e.g. private weight loss schemes), any drugs to help with weight loss (including orlistat, liraglutide and semaglutide), informal care/support and workforce participation will be piloted. These data will be collected from participants using a bespoke resource use questionnaire, which has been informed by the Database of Instruments for Resource Use Measurement (www.dirum.org) and reviewed by Patient Advisory Group members. Responses to the resource use questionnaire will indicate whether participants have difficulties understanding or completing the questions. Categories more prone to missing data will be reported and strategies developed to address this in a definitive trial (for example, using resource logs to improve the completeness of data [43, 44]).

The potential of the group-based intervention to impact on health-related QOL during the trial will be measured using EQ-5D 5L, which will be used to calculate quality-adjusted life years (QALYs) using the preference-based, UK tariff derived from members of the general public [36]. Information on well-being, measured using ICECAP-A, [45] will also be collected. The feasibility study will explore the extent to which EQ-5D 5L and ICECAP-A correlate with hypothesised mediator variables of PROGROUP effectiveness, such as shared social identity [46], which will inform the decision on whether to retain ICECAP-A in a definitive trial as a secondary outcome measure for economic evaluation.

#### Piloting the modelling framework

Assessment of the cost-effectiveness in the definitive trial will involve a within-trial economic evaluation and a ‘lifetime’ model-based economic evaluation, to explore the cost-effectiveness of PROGROUP compared to usual care. In the feasibility work, the modelling framework will be piloted to ensure that data collection methods are able to adequately capture patient and economic information required to inform the long-term extrapolation of clinical outcomes, costs and QOL data. In addition, the modelling framework will explore uncertainties and sensitivities in the evidence base using a ‘what-if analysis’ approach.

### Participant withdrawal

#### Withdrawal from treatment

The risk of harm being caused by the PROGROUP intervention is considered to be very low, as it is for usual care. However, we consider that there is a small potential risk of some participants requesting withdrawal for psychological reasons (e.g. uncomfortable with the group-based format of the PROGROUP arm), or at the discretion of the responsible clinical team. Participants may also choose to withdraw from the PROGROUP intervention at any time. Any intervention participant who withdraws will be asked to provide a reason but will be made aware that they are under no obligation to provide a reason, and that their withdrawal from the PROGROUP programme shall in no way affect their access to ongoing treatment.

Intervention facilitators may also choose, at their discretion, to withdraw participants from the PROGROUP programme. Grounds for withdrawing participants from the PROGROUP programme may include, for example, disruptive behaviour or willful non-engagement. Where possible, participants withdrawn due to disruption of group activities will be offered 1-to-1 alternative treatment.

Withdrawal from the PROGROUP programme, and the reason if provided, will be clearly documented in the participant’s clinical records and reported to PenCTU using a specific treatment discontinuation case report form.

Withdrawal from treatment does not preclude the participant from remaining in follow-up for research purposes. All participants withdrawn from PROGROUP (or usual care) will be encouraged to continue with trial follow-up visits and assessments as per protocol.

#### Withdrawal from follow-up

Participants may choose to withdraw themselves from the trial (whether in the PROGROUP or usual care arm) at any stage of the trial. Participants will be asked to provide a reason for withdrawal from follow-up but will be made aware that they are not obliged to give a reason and that their decision to withdraw will not affect their ongoing treatment. Participants who withdraw from follow-up will be contacted by a researcher to see if they are willing to discuss reasons for withdrawing. Withdrawal from trial follow-up and the reason, if known, will be clearly documented in the participant’s clinical records and reported to PenCTU using a specific trial withdrawal case report form. Data collected prior to withdrawal from follow-up will be included in the study analysis. Withdrawn participants will not be replaced with new participants.

### End of trial definition

Participants will complete their involvement in the trial after approximately 12 months post-randomisation, at the 12-month follow-up assessment. The trial will end on completion of all data collection.

### Adverse event reporting

The likelihood of participants being harmed by either the PROGROUP intervention or any of the trial procedures is very low. As such, the collection and reporting of adverse events in this trial will be restricted to only those events which meet the criteria for serious adverse events (SAEs). All unplanned hospital admissions detected will be reported, including admission to the Emergency Department; elective procedures will not be reported. Recorded from the time of randomisation until the end of trial visit, the primary means of detecting SAEs shall be interactions between the research team member(s) and the trial participant at each of the data collection timepoints. Detection of hospitalisations can also occur via participant self-report, as part of the health and social care resource use survey at each of the follow-up timepoints.

### Statistical analysis

To inform progression to the definitive trial, recruitment and completeness of key outcome data will be reviewed at 6 months post-randomisation. Final statistical analysis will be undertaken once all participants have completed the last assessment at 12 months post-randomisation and the trial database is locked. A statistical analysis plan (SAP) will be drafted by the trial statisticians, following CONSORT guidance for pilot and feasibility studies [47]. The SAP will be reviewed by the PSC and signed off by an independent statistician prior to database lock.

The flow of participants through the study will be presented in a CONSORT-style diagram with reasons for discontinuation or withdrawal given where available and rates of screening, enrolment, recruitment, randomisation and retention reported with 95% confidence intervals.

Descriptive statistics of participants’ demographic and baseline characteristics will be presented overall and by an allocated group to informally check for balance between groups and provide an overview of the study sample.

As a feasibility trial, the study is not powered to test the effectiveness of the intervention. Analyses of clinical and participant-reported outcomes will therefore be descriptive. Appropriate plots will be used to illustrate key data and assess for potential between-group differences but no formal, inferential statistical comparisons or hypothesis testing between groups will be undertaken.

For the participant-reported measures of eating behaviour, physical activity, capability, well-being, anxiety/depression, and social identification, variables will be derived according to published guidance and coding for which will be carried out independently by two statisticians. All clinical and participant-reported outcomes will be summarised at each time point, using descriptive statistics (e.g. numbers and percentages, means and standard deviations) alongside appropriate confidence intervals (taking into account the partial clustering in the intervention arm using mixed effects models). Changes between baseline and 6 months, and between baseline and 12 months, will also be summarised descriptively and presented by the allocated group on an intention-to-treat basis, with participants analysed in the group to which they were originally allocated.

Intervention engagement measures (e.g. number, length and frequency of sessions) will be descriptively summarised and reported.

The timing and frequency of missing outcome data will be summarised. As this is a feasibility study, no imputation of missing data will be undertaken, with the exception of instances where there are published methods for imputing missing items within a validated participant-reported outcome measure. Individuals lost to follow-up will be compared to those who complete the feasibility study to identify any potential bias, by means of descriptive statistics, but again with no formal hypothesis testing being undertaken. All statistical analyses will be undertaken using STATA version 16 or later, supplemented where required by R.

### Safety data

Safety data will be presented on a per-protocol basis. Serious adverse events will be cross-tabulated by group and assessed for clinical relevance.

### Data handling and record keeping

Data are collected and stored in accordance with the UK Data Protection legislation including the UK Data Protection Act 2018 and the General Data Protection Regulation, 2018. Each participant will be allocated a unique study number and is identified in all study-related documentation by their study number and initials. Data collected during the study and exported to the trial statistician, health economist and process evaluation specialists for analysis will be pseudonymised by the use of this unique identifier.

A web-based application developed and maintained by PenCTU will be used for trial management and for recording of quantitative participant data. This consists of a bespoke system for managing patient screening and participant randomisation, hosted on Microsoft Azure servers located in the UK, integrated with an electronic case report form (eCRF) built in REDCap Cloud hosted at the University of Plymouth [48, 49].

Digital recordings of audio data from qualitative interviews and session delivery will be stored on Microsoft SharePoint on the University’s secure server using the participant’s unique study number, accessible to the research team on a user permissions basis. Transcription of audio recordings of interviews or sessions will only be carried out by members of the research team or professional services with confidentiality agreements in place. During the study, members of the PenCTU and study team will have access to the dataset, on a user permission basis.

Access to the dataset will be granted to the Sponsor and host institution on request, to permit study-related monitoring, audits and inspections. After the programme has been reported, individual participant data that underlie the results will be available on request, in an anonymised form, along with supplementary files as required. Data will be shared with requestors whose proposed use of the data has been approved by the chief investigator and Sponsor, under an appropriate data sharing agreement.

### Governance

The study Sponsor, University Hospitals Plymouth NHS Trust, Plymouth, PL6 5FP, UK, assumes overall responsibility for the initiation and management of the trial. The Sponsor and funder will not have direct involvement in trial design, conduct, data analysis and interpretation, manuscript writing, and dissemination of results.

The trial was designed by the co-Chief Investigators and co-applicants (including patient representation) with support from the NIHR Research Design Service and the Peninsula Clinical Trials Unit.

Day-to-day trial management is administered through the UKCRC-registered Peninsula Clinical Trials Unit (PenCTU) at the University of Plymouth. PenCTU will conduct central and site monitoring in accordance with a risk-based monitoring plan and the Sponsor may audit trial conduct as deemed appropriate.

The Trial Management Group meets at least monthly to monitor the progress of the trial against the feasibility outcomes; review participant safety data; and address any issues that may arise. Independent oversight is provided by the PROGROUP Programme Steering Committee, comprising expert clinicians in patient weight management services, a statistician, a health economist, a health psychologist and patient representatives. The Steering Committee meets at least twice a year to oversee the conduct of the trial, and to monitor safety, ethical issues and data quality and completeness. A separate Data Monitoring Committee was not considered necessary for this feasibility trial.

For any amendment to the study, the Chief Investigator or designee, in agreement with the Sponsor, will submit information to the appropriate body in order for them to issue approval for the amendment. The Chief Investigator or designee will work with sites (R&D departments at NHS sites as well as the study delivery team) so they can put the necessary arrangements in place to implement the amendment to confirm their support for the study as amended.

### Public and patient involvement

PPI input has been provided by our PPI co-applicant (SP) and Patient Advisory Group (PAG), with independent PPI representation on the Steering Committee. The PPI co-applicant has been directly involved in the study since its inception. The PAG, led by the PPI co-applicant, has advised on protocol development and study design. This includes reviews of all patient-facing written material, aspects of data collection, selection of outcome measures, and the design of the qualitative study including the development and refinement of the topic guide. PPI representatives will also have a role in the analysis of data arising from this trial and the dissemination of results. If this feasibility trial is successful, the PPI group will play a central role in designing the definitive RCT proposed.

## Discussion

There is a need to develop the evidence base for the non-surgical management of severe obesity, and to determine the effectiveness and cost-effectiveness of interventions. However, the development and delivery of multi-centre clinical trials involving group-based behavioural interventions for the treatment of severe obesity presents a series of unique challenges, typified by experience of stigma, high prevalence of psychological drivers, previous poor experiences of care, frequent poor mobility, difficulties with access and attitudes to group care, and for this reason it was considered that a two-arm randomised controlled feasibility trial would be essential. In addition, given recent and upcoming changes in pharmacotherapy availability, data on the use of prescription drugs will add valuable context to inform future trial planning.

This feasibility study is intended to inform decisions about the design and delivery of a definitive trial and, accordingly, is not statistically powered to provide quantitative evidence of intervention effectiveness. The definitive trial will proceed if (a) progression criteria (including the number of sites able to run the intervention; proportion of participants attending; trial recruitment rate; sites can supply weight data for participants) are met or strategies can be developed to ensure they can be met in a definitive trial according to ‘stop-go’ green-amber-red criteria; (b) recruitment and retention rates, as predicted by the feasibility study, suggest acceptability of the trial procedures, including the randomisation process, and are sufficient for trial delivery within timescale.

## Supplementary Information

**Additional file 1.** Tabulated summary of feasibility outcomes.

**Additional file 2.** SPIRIT 2013 checklist.

## Data Availability

Not applicable.

## Abbreviations

AE: Adverse event
AR: Adverse reaction
CI: Chief Investigator
CRF: Case report form
PenCTU: Peninsula Clinical Trials Unit
DMC: Data Monitoring Committee
eCRF: Electronic case report form
GCP: Good Clinical Practice
ICF: Informed consent form
ISF: Investigator Site File (This forms part of the Trial Master File)
ISRCTN: International Standard Randomised Controlled Trials Number
NHS R&D: National Health Service Research & Development
NICE: National Institute for Health and Care Excellence
PI: Principal Investigator
PIS: Participant Information Sheet
PSC: Programme Steering Committee
QA: Quality assurance
QC: Quality control
QOL: Quality of life
RCT: Randomised controlled trial
REC: Research Ethics Committee
SAE: Serious adverse event
SAR: Serious adverse reaction
SDV: Source data verification
SOP: Standard operating procedure
SSI: Site Specific Information
SUSAR: Suspected Unexpected Serious Adverse Reaction
T3WMS: Tier 3 Weight Management Services
TMF: Trial Master File
TMG: Trial Management Group
UKCRC: UK Clinical Research Collaboration
