# Supplementary material for: A group-based behavioural intervention for weight management (PROGROUP) versus usual care in adults with severe obesity: a feasibility randomised controlled trial protocol
Source: Pilot Feasibility Stud. 2022 Sep 10;8:206. doi: 10.1186/s40814-022-01167-0 (PMC9463813; doi:10.1186/s40814-022-01167-0)
Supplement: Supplementary file 1 — Additional file 1. Tabulated summary of feasibility outcomes. [file 40814_2022_1167_MOESM1_ESM.docx]

#### Additional file 1. Tabulated summary of feasibility outcomes

| **Feasibility objectives** | **Outcome measures for feasibility assessment** |
| --- | --- |
| Rates of recruitment | Number of patients screened, consented and randomised (as a proportion of patients screened). |
| Rates of retention | Number attending follow-up visits. |
| Batch randomisation rate | Time required to recruit sufficient participants to trigger randomisation. |
| Data completeness and acceptability of outcome measures | Number of completed self-report questionnaires and missing items within each questionnaire. |
| Acceptability of planned approach for longer-term follow-up | Consent rates for additional follow-up data after the trial. |
| Fidelity of PROGROUP training and delivery | Fidelity assessment |
| Acceptability of trial processes and the intervention to participants | Attendance rates and intervention engagement. |
| Adherence to usual care | T3WMS attendances. |
| **Participant data collection items** | **Outcome measures proposed for future definitive RCT.**  **Measured as change from baseline at 6 and 12 months post randomisation** |
| Weight loss | 1. Weight in kilogrammes (proposed primary outcome and primary endpoint).  2. Percentage of participants achieving ≥5%* and ≥10% weight loss at 6 and 12 months.  3. Body Mass Index (BMI). Calculated from height at baseline and weight at the time-point. |
| Glycaemia measurement | HbA1c |
| Blood Pressure | Mm Hg |
| Lipid profile | Total Cholesterol, HDL Cholesterol, Triglycerides |
| Alcohol use | Alcohol units |
| Eating behaviour | Adult Eating Behaviour Questionnaire |
| Physical activity | IPAQ short form |
| Health-related quality of life | EQ-5D-5L |
| Well-being | ICECAP-A, PHQ-4, Self-esteem and life satisfaction measures. |
| Health, social care and wider societal resource use | Resource use questionnaire |
| Co-morbidities | Reported comorbidities |
| Medication use | Reported relevant medications |
| Social identification** | Social identification measure |
| Loneliness** | Loneliness measure |

*Minimum clinically worthwhile weight loss.

** Also change at 3 month point (mid-intervention)
